# Carbon monoxide to ‘simulate altitude’: The ethical grenade

**DOI:** 10.1113/EP093704

**Published:** 2026-02-27

**Authors:** Raphael Faiss, Franck Brocherie

**Affiliations:** ^1^ Research and Expertise in antiDoping sciences (REDs), Institute of Sports Sciences University of Lausanne Lausanne Switzerland; ^2^ Laboratory Sport, Expertise and Performance (EA 7370) French Institute of Sport (INSEP) Paris France

1

We read with interest the study reporting the ‘Effect of live‐high, train‐low strategy induced by chronic low‐dose carbon monoxide exposure on haematological parameters and performance in trained individuals’ (Villanova et al., 2026). The investigators should be commended for conducting a challenging study with athletes exposed to two conditions (with or without carbon monoxide (CO)) 6 months apart. They intended the study ‘solely for the scientific community’ in light of the current prohibition by the World Anti‐Doping Agency (WADA) of the use of CO by athletes or their entourage in force since 1 January 2026. An increase in total haemoglobin mass (tHb_mass_) is reported after their CO exposure protocol, but we argue it is inefficient for athletes aiming to improve performance. Endurance athletes generally embrace various forms of training, including altitude/hypoxic strategies, in their preparation for peak performance. The eventual goal is an improved aerobic exercise capacity observed even in elite athletes, despite even trivial changes in tHb_mass_ (Krumm et al., 2024). The primary mechanistic understanding of performance improvement was purportedly linked to an augmented tHb_mass_ improving oxygen convection. Recent evidence, however, underlines that the sole increase in tHb_mass_ is insufficient to improve endurance performance; the latter increase may be due to a natural ‘live high–train high’ camp (Cubel et al., 2026) or altitude exposure combined with erythropoietin administration (Bonne et al., 2025).

Altitude training therefore requires, by definition, a careful balance between the effects of altitude level/hypoxic dose (for putative increase in tHb_mass_) and the aims of training (for better performance and other accompanying hypoxic‐ and exercise‐induced physiological adaptations), so that blood manipulation interventions moving only the cursor of tHb_mass_ cannot be recommended in the absence of performance improvement, like in the study by Villanova et al. (2026). A further look at their results underlines, for instance, absolutely no performance benefit, whereas they framed and contextualized their article toward a proof‐of‐concept performance enhancing method challenging traditional altitude/hypoxic training models. Besides, blood markers support a deceleration of the erythropoiesis (lower immature reticulocyte fraction) with lesser bone marrow activity (lower medium fluorescence ratio). From an anti‐doping perspective, considering the small sample size and the known large individual variability, the trend observed for an increased OFF‐Score points toward individual flags that WADA's Athlete Biological Passport approach would certainly have identified. Detailed individualized profiles from their study would certainly have allowed outlining this and unequivocally highlighted its prohibited practice.

In summary, if athletes were to use repeated CO administrations to increase their tHb_mass_, it would probably be identified in the current anti‐doping framework, and it would probably not improve their performance. As researchers active with elite athletes but linked to anti‐doping organizations, we believe that the narrative choice emphasizing performance‐related outcomes inadvertently legitimizes applicability of an approach that has been clearly excluded from sport.

The proposed ergogenic mechanism requires a toxic exposure. Unlike hypoxic tents or altitude exposure, CO creates a poisoning physiology (COHb) rather than a ‘natural’ environmental hypoxia. Even ‘low‐dose’ CO protocols are deliberately trying to reach a biologically meaningful COHb, meaning users are intentionally inducing a state that public‐health recommendations work to prevent. A target of 15% COHb is very closed to the lethal toxicity threshold, considering CO is odourless and colourless for which the dose inhaled is difficult to evaluate. The risk is not just ‘acute CO poisoning’; it is error‐prone dosing and delayed harm, with the evidence that a distinctive hazard with CO is the possibility of delayed neurocognitive effects after apparent recovery (Zhang et al., 2024). Even if rare at lower COHb, the ethical burden is that sports settings often encourage repetition and escalation (‘if some is good, more is better’, especially in vulnerable athletes). The risk profile of using CO includes high‐consequence outcomes and delayed neurological harm. It is insufficient to mention that CO use is now prohibited as blood manipulation, because it is not ethically or medically justifiable. Overall, the lack of CO‐induced performance benefit and the existence of lower‐risk alternatives with established altitude/hypoxic training strategies should be discussed with the athletes, emphasizing the real danger associated with CO use. In the high‐pressure environment of elite sport, prone to dose escalation and imperfect supervision, the real‐world sports environment is definitely not as controlled as laboratories.

Overall, any uncertain or non‐existent performance gain does not justify introducing a known toxicant whose harms include acute hypoxic injury and potentially delayed neurological effects.

Finally, Villanova and colleagues, by showing the inefficacy of repeated CO exposures to improve performance (despite a significant biological effect actually known for decades), may allow us to close the toxic discussion on CO use by athletes. Future work in this area must definitely adopt an explicit and unambiguous position, clearly dissociating (physiological) mechanistic exploration from any performance‐enhancement application, in particular when a method aligns with the criteria of a prohibited substance or method, whether already banned or not. Such an approach would preserve the scientific value of the research while avoiding ethical ambiguity and potential misinterpretation by practitioners, coaches or athletes. This may also help re‐centre research efforts on altitude/hypoxic‐training methodologies that genuinely promote performance through haematological and non‐haematological pathways, rather than relying on toxicological surrogates such as CO.

## AUTHOR CONTRIBUTIONS

All authors have read and approved the final version of this manuscript and agree to be accountable for all aspects of the work in ensuring that questions related to the accuracy or integrity of any part of the work are appropriately investigated and resolved. All persons designated as authors qualify for authorship, and all those who qualify for authorship are listed.

## CONFLICT OF INTEREST

R.F. is member of the Polish Anti‐Doping Agency's (POLADA) scientific committee. F.B. is member of the French Anti‐Doping Agency's (AFLD) scientific committee.

## FUNDING INFORMATION

Raphael Faiss did not receive any fund. Franck Brocherie received fund through the Laboratory Sport, Expertise and Performance (EA 7370), which is a partner of the French‐speaking network ReFORM, recognized as a Research Centre for the Prevention of Injury and Illness and the Protection of Athletes by the International Olympic Committee (IOC). As a member of the IOC Medical Research Network, ReFORM has received funding from the IOC to establish long‐term research programs on the prevention of injuries and illnesses in sport for the protection of athlete health.
